# Evidence for Pituitary Repression of the Human Growth Hormone-Related Placental Lactogen Genes and a Role for P Sequences

**DOI:** 10.3390/ijms26094421

**Published:** 2025-05-06

**Authors:** Peter A. Cattini, Yan Jin

**Affiliations:** Department of Physiology and Pathophysiology, University of Manitoba, Winnipeg, MB R3E 0J9, Canada

**Keywords:** transcription, tissue specificity, chromatin, histones, pituitary, growth hormone

## Abstract

The human (h) growth hormone (GH)/placental lactogen (PL) gene family has served as an important model to study tissue-specific expression. The two GH genes (*hGH-N*/*GH1* and *GH-V*/*GH2*) and three PL or chorionic somatomammotropin hormone (CSH) genes (*hPL-L*/*CSL1*, *hPL-A*/*CSH1* and *hPL-B*/*CSH2*) are clustered together at a single locus. Although they share >90% sequence similarity, *hGH-N* is expressed by somatotrophs of the anterior pituitary while the remaining four hGH/PL genes are expressed by the villous syncytiotrophoblast of the placenta. Efficient pituitary expression depends on a locus control region (LCR) that includes nuclease hypersensitive sites I-V (HS I-V). For activation, data indicate that HS III facilitates the initial access of pituitary-specific transcription factor Pit-1 to the locus, where it is required to bind Pit-1 sites at HS I/II and the *hGH-N* promoter. This is associated with histone acetylation and tri-methylation modifications that are consistent with active chromatin. However, all five hGH/PL genes share similar nuclease sensitivity in human pituitary chromatin, suggesting similar levels of accessibility and thus potential for transcription. Furthermore, *hPL-A* and *hPL-B* promoters contain Pit-1 binding sites, and the *hPL-A* promoter, like *hGH-N*, will support expression in transfected pituitary tumor GC cells in culture. These observations suggest the possibility of a transcriptional repressor mechanism that prevents hPL gene expression in the pituitary. P sequences were identified as a candidate. They are located upstream of all four placental hGH/PL genes but not *hGH-N*, repress *hPL-A* promoter activity in transfected pituitary GC cells, and bind a forkhead box A1/nuclear factor-1 transcription, which is proposed to act as a repressor complex in human pituitary chromatin. In spite of this, the inability to limit *hGH-N* expression when tested in transgenic mice brought the role of P sequences in pituitary repression into question. These observations are re-examined here in light of new evidence that the LCR (HS III) interacts with P sequences in the human pituitary.

## 1. The Human Growth Hormone and Placental Lactogen Genes

**Figure 1:**
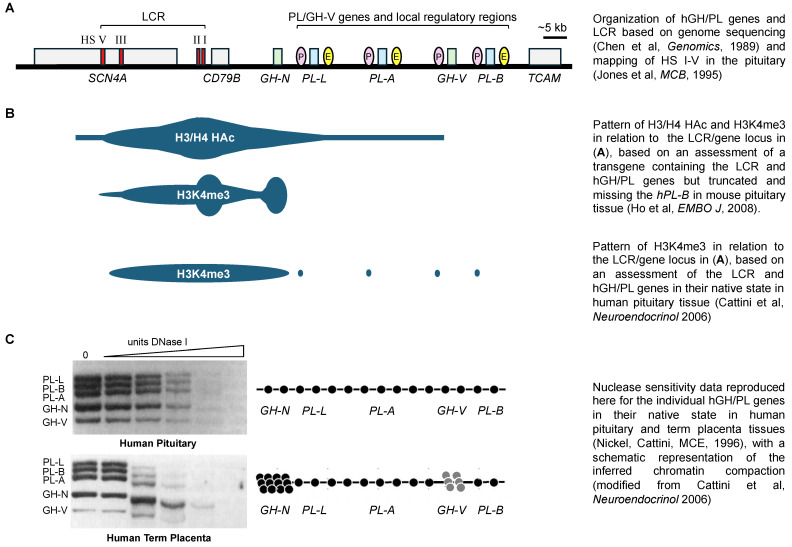
(**A**) Schematic representation of the human (h) GH/PL locus control region (LCR) and genes, as well as nearby skeletal muscle-specific sodium channel SCN4A, lymphocyte-specific CD79B and testis-specific TCAM genes located on chromosome 17 [7,8,9]. The relative positions of LCR hypersensitive sites (HSs), P sequences (P) and downstream enhancer sequences (E) are shown. HS I and II are pituitary-specific, while HS III and V are found in both the pituitary and placenta. HS IV is placenta-specific and not shown. (**B**) Summary of histone modifications in relation to the LCR and gene locus shown in (**A**), including combined histone H3/H4 acetylation (H3/H4 HAc) [10] and tri-methylation at lysine 4 of histone H3 (H3K4me3) [10,11,12]. (**C**) Autoradiograph showing previously reported results of a nuclease assay of DNA accessibility of hGH/PL genes in chromatin from human pituitary and term placenta [13], and a schematic representation of the outcome [11]. In the pituitary, all five gene fragments are equally sensitive to DNase I digestion, suggesting that the chromatin structure containing the placental hGH/PL genes is decondensed and similar to the actively expressed *hGH-N*. In the placenta, the fragment containing *hGH-N*, which is not expressed, and to a lesser extent *hGH-V*, which is minimally expressed, are more resistant to DNase I digestion, and are thus depicted with a more condensed nucleosomal organization compared to the hPL genes.

While somatic cells of a multi-cellular organism contain the identical complement of genes, not all genes will be expressed in the same tissues or cells at the same time. Precise regulation is required for spatial and temporal-specific gene expression during development and to ensure physiological function. The human (h) growth hormone (GH) gene family has proven to be an important and intriguing model for understanding gene regulation, given their structural similarity and expression in two distinct tissues and independent cell lineages. The five members of the hGH gene family arose through three gene duplications and one gene deletion event likely 15 million years ago [1]. As a result, these genes share greater than 90% sequence identity in their coding and upstream flanking DNA [1], and are clustered together in the same transcriptional direction at a single locus of 47 kilobases (kb) on the long arm of chromosome 17 (q22-q24) [2] (Figure 1A). The family consists of the pituitary GH gene (*hGH-N*/*GH1*) and the placental GH gene (*GH-V*/*GH2*), two genes (*hPL-A*/*CSH1* and *hPL-B*/*CSH2*) that independently code for placental lactogen (PL), which is also referred to as chorionic somatomammotropin hormone (CSH) [3], and the hPL-L pseudogene (*hPL-L*/*CSHL1*) [1,2]. The hGH/PL genes have proven to be an important and intriguing model for understanding gene regulation, given their structural similarity and expression in two distinct tissues and independent cell lineages. The hGH-N gene is expressed preferentially by the somatotrophs of the anterior pituitary, and a GH gene is present in all vertebrates. By contrast, the PL genes in primates, including humans, are expressed by the syncytiotrophoblast of the placenta, which is derived from the blastocyst during pregnancy [4,5]. Furthermore, the primate PL genes, like *GH-V*, are structurally related to the GH-N gene as opposed to the prolactin gene; this contrasts with rodents and other vertebrates where the PL genes are highly related to prolactin [2,6].

The hPL locus control region (LCR) includes three hypersensitive sites (HS III-V) [14,15]. HS III possesses enhancer activity and binds activator protein (AP)-2α [14], while HS IV, which is placenta-specific, and HS V are linked to locus boundary element activity through association with CCCTC binding factor (CTCF) [14,15,16,17,18]. Regulatory regions near the hPL genes, such as upstream P sequences and the downstream 3′-regulatory region (3′-RR), also play roles in modulating gene expression by interacting with multiple transcription factors [2,19,20,21,22,23,24,25]. However, no transcription factor that limits hPL locus activation and presumably LCR/gene interactions in the transitioning CTB has been reported, but the paternally expressed gene 3 (PEG3/PW1) transcription factor is a candidate [26].

PEG3 is a zinc finger transcription factor expressed from the paternal allele that binds the consensus sequence 5′-gkGGswsT-3′ site with high affinity and regulates multiple cellular processes, potentially as a transcriptional repressor [26,27,28,29,30,31]. Putative PEG3 binding sites are present in both the hPL LCR and proximal promoter regions [26]. Furthermore, PEG3 is expressed in various villous CTB subtypes, including syncytin-2-positive CTBs that fuse to generate the multinucleated syncytiotrophoblast layer, but not by the syncytiotrophoblast itself [32,33,34,35]. Together, these observations suggest that PEG3 plays a role in limiting hPL gene locus activation and/or expression.

Here, we investigated PEG3’s ability to bind the hPL LCR and promoter sequences in CTB-like JEG-3 cells and term placentas, where variations in the levels of hPL gene expression by maternal obesity and gestational *diabetes mellitus* (GDM) are reported [36]. Our findings support a role for PEG3 in the regulation of hPL gene expression by repression and/or by limiting the activation of the hPL locus, which requires a specific chromosomal architecture that is disrupted with maternal obesity and associated with ongoing or increased PEG3 binding at HS IV. Schematic representations of these regulatory interactions are presented.

## 2. The hGH/PL Gene Cluster and the Role of Pit-1

The chromatin structure and accessibility of specific regulatory regions that support transcription factor complexes play essential roles in the efficient expression of the hGH/PL genes, and distinct mechanisms are involved in the pituitary and placenta [11,13,18,37,38]. The pituitary expression of *hGH-N* in vivo relies on regulatory sequences associated with nuclease hypersensitive sites (HS I-V), which make up the hGH locus control region (LCR) [18] (Figure 1A). Hypersensitive sites represent highly accessible DNA regions in the chromatin that may allow the entry of factors and complex formation and/or facilitate the further modification of the chromatin structure. HS I and II are pituitary-specific and located about 14.6 kb upstream of *hGH-N* in the upstream flanking DNA of the lymphocyte-specific CD79B gene. The three remaining sites, HS III, HS IV and HS V, are located 28, 30 and 32 kb upstream of *hGH-N* in the skeletal muscle-specific sodium channel SCN4A gene [39]; HS IV is placenta-specific while HS III and V are detected in both the pituitary and placenta [18]. The entire LCR encompassing HS I-V was required to recapitulate pituitary-specific *hGH-N* expression in transgenic mice [18]. HS V is believed to serve a boundary function for the *hGH-N* locus, which is consistent with its enhancer-blocking activity and ability to bind the CTCF insulator protein [16]. The HS I/II sequences contain three Pit-1 sites [40], which bind Pit-1 and, together with Pit-1 binding to the proximal *hGH-N* promoter region, work in concert to activate *hGH-N* in the pituitary and act as a potent enhancer [40,41]. HS III is proposed to serve as an entry point to facilitate access by Pit-1 to the LCR and binding at HS I/II sequences with the appearance of Pit-1 during somatotroph development [42,43]. This mechanism may include the ability of Pit-1 to bind through protein–protein interaction, increase RNA polymerase activity and enhance the generation of non-coding transcripts tracking outwards, thereby initiating the chromatin remodeling required to provide HS I/II access and further structural change [43]. Thus, in terms of transcription factors, Pit-1 is essential for efficient pituitary *hGH-N* expression.

However, although not expressed in the pituitary, the *hPL-A* and *hPL-B* proximal promoter regions possess Pit-1 sites at equivalent locations to those found in the *hGH-N* proximal promoter region, which are able to bind Pit-1 [12,44]. The *hPL-A* promoter, including about 0.5 kb of upstream flanking DNA, will also direct hybrid reporter gene expression in transfected pituitary tumor cells in culture as effectively as equivalent *hGH-N* promoter sequences [12,45]. Furthermore, for both *hPL-A* and *hGH-N*, this activity is lost when their Pit-1 sites are disrupted [45]. The question then arises as to whether there is any evidence that the hPL genes are accessible to transcription factor binding in the context of chromatin in the human pituitary.

## 3. LCR and hGH/PL Gene-Related Sequence Interactions in Pituitary Expression

In spite of the presence of Pit-1 sites in the proximal promoter regions of the hPL genes, their ability to function would require them to be accessible in the human pituitary. The physical access of transcription factor complexes to DNA, including RNA polymerase II-related complexes, is facilitated by the de-condensation of the chromatin in which the DNA or genes are packaged [46]. In brief, chromatin consists of DNA wound around a disc-shaped nucleosome core composed of two copies each of histones H2A, H2B, H3 and H4, and in this form (often referred to as a 10 nm fiber), genes are transcriptionally active or poised to be transcriptionally active, with features of both gene activation and repression [47]. However, histones from adjacent nucleosomes can interact through the unstructured (tail) regions of these core histones as well as (linker) histone H1, and condense the chromatin into irregular and regular higher order or heterochromatin structures that are not active depending on conditions [48,49,50]. Thus, the posttranslational modification of histones, including H3 and H4 acetylation and methylation, has the potential to favor a de-condensed accessible or condensed inaccessible chromatin structure [50,51]. In general, histone acetylation occurs at lysine residues and it increases the potential for gene expression, while histone methylation may activate or repress gene expression depending on the residue methylated [50]. For example, the tri-methylation of lysine 4 of histone H3 (H3K4me3) is associated with active chromatin, while the tri-methylation of lysine 27 (H3K27me3) is linked to inactive heterochromatin formation [52]. HS I, II, III and V are re-established in the pituitaries of mice containing the hGH LCR and four of the five hGH/PL genes (*hGH-N*, *hPL-L*, *hPL-A* and *hGH-V*) as a transgene, even though this transgene did not include *hPL-B* [18,41]. The combined levels of acetylated histone H3 and H4 were assessed across the hGH/PL gene locus in the pituitaries of these mice by chromatin immunoprecipitation (ChIP) using antibodies for acetylated histone H3 and H4 (H3/H4 HAc) [38]. The ratio of bound (immunoprecipitated) to unbound DNA for each site assessed was normalized to the corresponding ratio obtained by rehybridizing the membrane with a total mouse genomic DNA probe to control for DNA loading on the hybridization membrane, and this total genomic acetylation ratio (Bound/Unbound) was defined as 1.0 [38]. Thus, a Bound/Unbound ratio above 1.0 is suggestive of increased acetylated chromatin and thus the potential for a more open/active configuration. A peak of acetylation with a Bound/Unbound ratio of ~10 was detected in the region containing HS I/II [38]. This extended out in both directions to include HS V sequences and the *hGH-N* promoter region, with a Bound/Unbound ratio of ~3.7 compared to 1.0 for the total background genomic DNA [38]. However, a Bound/Unbound ratio of ~2.2 was also detected for sequences downstream of *hGH-N*, which contains *hPL-L* and *hPL-A* (schematically represented in Figure 1B). A similar pattern was also seen when acetylated histone H3 and H4 were assessed independently. The ratios for hGH-N and hPL gene-related sequences were above background in all cases. While values are undeniably low for the hPL gene-related sequences when compared to HS I/II, they are comparable to those detected for *hGH-N* [38]. Similarly, regions containing HS I-III and *hGH-N* promoter sequences were associated with H3K4me3; but again, at least in the human pituitary, H3K4me3 chromatin was also above the background for sequences that can be found in regions upstream of all four hPL/GH-V genes (Figure 1B). Thus, the pattern of histone modification as determined by ChIP assay using pituitaries from hGH/PL transgenic mice does not rule out the possibility that the placental genes and the *hPL-A* promoter are accessible to Pit-1 binding. Indeed, an assessment of the sensitivity of hPL/GH-V DNA to deoxyribonuclease in the human pituitary, described below, supports this possibility.

The DNA nuclease (DNase I) sensitivity assay is based on the idea that DNA in condensed chromatin would be relatively resistant to digestion by DNase I because of the more limited access to this ~38 kDa protein, in much the same way that it would be resistant to binding other protein or transcription factors. By contrast, DNA in the context of de-condensed chromatin (10 nm nucleosome fiber) would be more accessible and thus relatively more sensitive to nuclease digestion. The nuclease sensitivity of the hGH/PL gene locus in human term placenta and pituitary tissue samples was compared [13]. In placenta chromatin, the three hPL genes were significantly more sensitive to nuclease digestion when compared to *hGH-N* and to a lesser extent *hGH-V.* This is consistent with open and active hPL genes as well as the lack of *hGH-N* expression and the minimal expression of *hGH-V* in the placenta [53,54]. By contrast, in the human pituitary tissue sample, all five genes showed similar nuclease sensitivity [13] (re-presented in Figure 1C), suggesting that the condensation of the chromatin containing *hPL-A* and *hGH-N* is similar. As *hGH-N* is expressed, this further suggests that the *hPL-A* and the other hPL/GH-V genes are contained in a poised chromatin configuration [47]. Thus, while not transcriptionally active, the hPL/GH-V genes are potentially accessible to transcription factor binding based on the ability of DNase I to bind and digest their DNA [13]. However, this does not rule out the possibility that the absence or repression of hPL gene expression is related to a lack of LCR interaction.

## 4. HS I/II and HS III Interactions with *hGH-N* Are a Feature of Efficient Pituitary Expression

**Figure 2:**
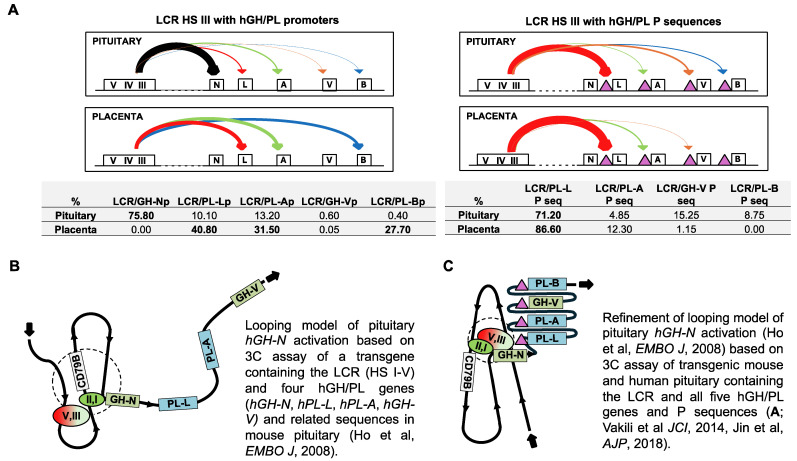
(**A**) The frequency of LCR (HS III) interaction with upstream hGH/PL gene promoter regions and upstream P sequences was assessed by 3C assay. The frequency of ligation between the HS III and promoter regions was reported previously [36]. Ligation products from pooled human pituitary (n = 3) and term placenta samples (n = 9) were amplified with specific primer pairs to detect interactions between the LCR and hGH/PL promoters and P sequences. A higher ligation frequency indicates that the DNA fragments associated with the regions investigated are in close proximity with each other in the cell nucleus in situ during the period of testing, even if separated by a large distance on the chromosome. Values are expressed as a percentage and normalized for glyceraldehyde 3-phosphate dehydrogenase DNA. The “total” interactions for each tissue were arbitrarily set to “100%” to allow comparisons, and the values for the percentage of all interactions assessed are tabulated. The tabulated results are also presented graphically, and the arrow thickness gives an approximation of the relative frequency of interaction. Arrows are coloured to assist with a comparison of the interaction, as assessed in the pituitary versus placenta. Schematic representations of the interactions involved in the activation of *hGH-N* in the pituitary (broken circle) based on the chromatin conformation capture (3C) data in (**B**) a mouse model with LCR and four hGH/PL genes as a transgene and using either HS II or sequences common to all four hGH/PL genes as anchors for 3C assay [10]; and (**C**) a mouse model with LCR and all five hGH/PL genes as a transgene [55], as well as human pituitary using HS III as the anchor for 3C assay [36], in addition to information in the study used for (**B**).

Elegant transgenic mouse studies combined with observations made using the chromatin conformation capture (3C) assay have provided insight into the sequence interactions required for hGH gene activation and expression. Briefly, the 3C assay allows the detection of regions of DNA that, although separated by tens of thousands of base pairs on a chromosome, are located in close proximity to each other in the context of the cell nucleus in situ through the folding of chromatin. This is reflected by a higher ligation frequency of the two regions in the 3C assay. Specifically, a 3C assay of transgenic mouse pituitary tissue revealed that *hGH-N* activation requires interaction between HS I/II and the *hGH-N* promoter, as well as the binding of Pit-1 at both sites [10,41]. A pituitary-specific interaction between HS II and HS III-V was also described in these mice [10]. Subsequently, a further interaction between HS III-V and the *hGH-N* promoter was identified in human pituitary tissue by a 3C assay [36] (reproduced as part of Figure 2A). Together, these observations indicate a complex series of interactions between sequences in the LCR and *hGH-N*, involving multiple chromatin loops [10] (Figure 2B,C). This suggests that a lack of hPL gene expression in the pituitary might be explained by the absence of similar loop structures and or a lack of interactions with sequences in the LCR. Although not identical, there is evidence, however, that the hGH LCR can interact with hPL/GH-V-related sequences in the pituitary. Sequences originally described as “p” were identified about 2 kb upstream of the placental hPL/GH-V genes but not pituitary *hGH-N* [2]. These P sequences are also associated with H3K4me3 (Figure 1B). The potential interactions between hPL/GH-V P sequences and HS III were assessed by a 3C assay in human pituitary tissue using the same approach described previously for human term placenta samples [39]. Multiple interactions were observed, including a major interaction between HS III, a proposed entry point for Pit-1 to the hGH locus [43], and *hPL-L* P sequences (Figure 2A). It is noted that the previous study in human term placenta samples also highlighted the potential importance of LCR interactions, including HS III with hGH/PL-related sequences, in establishing active/inactive gene domains [39].

**Figure 3:**
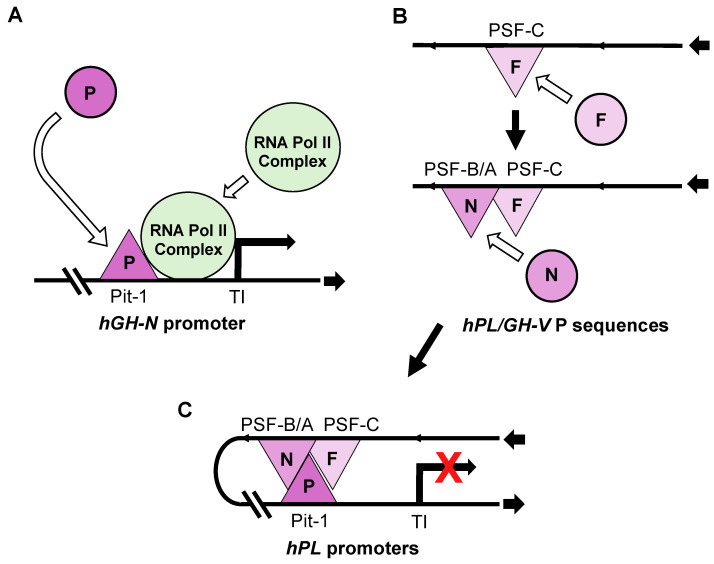
Conceptual model for pituitary repression of placental members of the human (h) GH/PL gene family suggested by nuclease protection, EMSA, immunoprecipitation and gene transfer data using rat pituitary tumor GC cells and human pituitary tissue [19,45,56,57,58,59]. (**A**) Pituitary-specific transcription factor Pit-1 (P) binds in the promoter region of all hGH/PL genes, including equivalent sequences at around nucleotide position −100/−60 of *hGH-N* and *hPL-A*, and is required for efficient promoter/RNA polymerase activity at the transcription initiation site (TI) in transiently transfected GC cells. (**B**) A 263 bp fragment of P sequences (263P) located about 2 kb upstream of placental hPL/GH-V genes will repress *hPL-A* promoter activity in transiently transfected GC cells. Three nuclease protected regions, PSF-A, B and C, can be identified in the 263P fragment. FOXA1 (F) binds at PSF-C, with the ability to interact with DNA regardless of chromatin condensation and support the recruitment of NF-1 (N), which binds at PSF-A/B. (**C**) In the presence of P sequence and promoter Pit-1 DNA, the FOXA1, NF-1 and Pit-1 participate in a common pituitary-specific repressor complex that can interfere with transcription.

In spite of this, it is unclear whether the absence of an interaction with the LCR, whether HS I/II or HS III, would be sufficient to completely block *hPL-A* or *hPL-B* expression in the pituitary. When transgenic mice were generated using only the *hGH-N* directed by ~0.5 kb of promoter sequences as the transgene and no LCR, *hGH-N* RNA was detected in the pituitaries of three of the five mouse lines tested. Again, *hPL-A* and *hPL-B* promoters are able to bind Pit-1 at equivalent sequences to those found in the *hGH-N* promoter [12,44], and ~0.5 kb *hPL-A* and *hGH-N* promoter sequences possess similar activity in transfected rat pituitary tumor cells in culture [45] (Figure 3A). Thus, the detection of the hPL gene RNA might be expected, given that based on nuclease sensitivity, hPL genes are contained in an open chromatin configuration in the pituitary and, presumably, in the absence of any suppressing sequences and/or factors [13]. However, there is evidence that repressor activity might act on hPL genes in the human pituitary.

## 5. P Sequences Bind Transcription Factors with Repressor Function in Human Pituitary Tissue

As a result of their location, it was suggested that P sequences may play a role in hPL/GH-V gene expression in the placenta [2]. However, no P sequence enhancer activity was detected when tested upstream of a hybrid *hPL-A* promoter–reporter gene in transiently transfected human choriocarcinoma cells [56]. There was also no consistent placental activation of *hPL-A* when P sequences were tested in their native context about 2 kb upstream in transgenic mice (23). However, the specific expression of *hPL-A* and *hPL-B* was seen in the mouse placenta when a transgene including all five hGH/PL genes and an intact LCR (HS I-V) was used [60]. More recent data suggest that P sequences may play a role in hPL gene domain activation in human placenta, and that this requires interaction with the LCR [39].

There is also evidence, however, that P sequences possess repressor activity in pituitary cells in culture [19,56,57,61], raising the possibility that these sequences play a role in suppressing hPL/GH-V gene expression in the pituitary. A 2.4 kb DNA fragment containing P sequences or a 263 base pair (bp) sub-fragment (263P element) repressed reporter gene activity directed by a 0.5 kb *hPL-A* promoter >90% in transiently transfected rat pituitary tumor GC cells [56]. Multiple transcription factor protein interactions with 263P DNA sequences were detected in vitro by a nuclease protection assay and further characterized by electrophoretic mobility shift assay (EMSA) using GC cell nuclear extract [19,56,57,59]. Initially, two regions of protein–DNA interaction were detected by nuclease protection assay, P sequence fragments (PSF)-A and B [56]. Subsequently, the protected region containing PSF-A was extended using enrichment through the heparin-agarose fractionation of the GC cell nuclear extract, revealing PSF-C [19,58]. The disruption of 263P or sub-fragments (including PSF-A, PSF-B and PSF-C) resulted in the decrease or loss of repressor activity, when tested on the 0.5 kb *hPL-A* promoter in GC cells [19,57,58,61]. As a result, a repressor complex was identified containing a member of the forkhead box (FOX) family of transcriptional regulators, FOXA1, also known as hepatocyte nuclear factor-3α (HNF-3α) [19]. FOXA1 interacts with nuclear factor-1 (NF-1) and can bind to P sequences in the context of human pituitary chromatin in situ [19,57].

A feature of the FOX family is a highly conserved 100 amino acid winged-helix motif that has a structure similar to linker histones H1 and H5 [62,63]. As a result, it is able to displace the linker histone, and thereby potentially decondense chromatin and allow other transcription factors to bind locally [62,64], including NF-1 that is unable to bind in condensed chromatin [64]. Members of the NF-1 family of transcription factors can activate or repress gene expression depending on the cell or tissue type and promoter context [65,66]. This would be consistent with a potential role for P sequences and NF-1 in both the placenta and pituitary. Thus, these observations suggest that FOXA-1 binds to P sequences in the human pituitary, regardless of chromatin compaction, which may facilitate the nearby recruitment and/or binding of NF-1, and the generation of a FOXA1/NF-1 complex (Figure 3B).

The mechanism of P sequence-related repression in the pituitary by the FOXA1/NF-1 complex is not defined and we cannot rule out the possible involvement of other factors that have been linked to repression; this includes, for example, NF-Y and GATA2/3 [67,68]. However, repression of the 0.5 kb *hPL-A* promoter activity in transfected pituitary GC cells was only detected in the presence of an intact Pit-1 site at nucleotide position −97/−66, relative to the transcription initiation site (+1) [19]. Furthermore, the interaction identified between HS III, as a potential entry point for Pit-1 binding, and P sequences suggests a possible mechanism for Pit-1 recruitment as a necessary component of the P sequence/hPL promoter repressor complex. Regardless, the need for an intact Pit-1 site in the *hPL-A* promoter suggested that interfering with Pit-1 complex formation and/or Pit-1-directed promoter activity at the hPL gene promoter is a likely target for P sequence-related repression, with Pit-1 itself providing pituitary specificity [19,56,59]. This was supported by the ability of hPL-A (−101/−62) and hGH-N (−100/−60) oligonucleotides, which contain equivalent promoter Pit-1 DNA elements, to complete FOXA-1/NF-1 binding to P sequences in vitro using GC cell nuclear extracts as a source of Pit-1 protein [56,59]. It is also noted that recombinant Pit-1 does not bind to the 41 bp PSF-C region containing the FOXA1 site [59], and in all cases where the indirect competition of Pit-1 was detected in nuclease protection and EMSAs, the 41 bp PSF-C region containing the FOXA1 site was required [56,59]. These observations are consistent with the participation of Pit-1, FOXA1 and NF-1 in a common repressor complex requiring binding to promoter Pit-1 DNA and P sequences, which presumably interferes with RNA polymerase recruitment and/or promoter activity. This is presented as a conceptual model in Figure 3C.

There is one reported attempt to test the P sequences for pituitary activity in a developmentally dynamic setting by using transgenic mice [21]. No repressor activity was detected but unfortunately the design of the transgene likely compromised the test. The 263P element was used and inserted immediately downstream of a 1.6 kb *Bgl*II fragment containing the HS I/II pituitary enhancer region, and immediately upstream of *hGH-N* with only ~0.5 kb of flanking and promoter sequences [21]. A second P element was also inserted downstream of *hGH-N* in the transgene. Thus, the effect of P sequences on hPL/GH-V gene promoter activity was not tested. Also, the 1.6 kb *Bgl*II fragment is a strong enhancer when used for hybrid gene transfer experiments in vitro and as part of a transgene in vivo [18,40]. In transgenes where the 1.6 kb *Bgl*II (HS I/II) fragment was inserted directly upstream of the 0.5 kb promoter-hGH N gene, expression was at least 50–100 times greater than when transgenes contained HS I-V and/or when the native distance (~15 kb) between HS I/II and *hGH-N* was maintained [18]. Thus, the high level of enhancer activity might have hindered the detection of any repressor mechanism as a result of the proximity of the component HS I/II, P and promoter sequences in the transgene as tested. This also assumes that the proximity did not hinder interaction between regulatory regions at the chromatin level (for example, limiting looping and/or complex formation), and that all the required regulatory regions were present. In this context, HS III and V sequences of the LCR were absent from the transgene used to test the 263P element. Based on a more recent 3C assay of human pituitary tissue, the evidence now suggests that the LCR and specifically HS III interacts with P sequences in the human pituitary (Figure 2A), supporting an alternative interpretation of the chromosomal architecture for the hGH/PL gene locus in the pituitary (Figure 2C).

In summary, the placental hPL/GH-V genes are not transcriptionally active but are contained in a nuclease sensitive chromatin configuration in human pituitary tissue. The ~0.5 kb hPL gene promoters are able to bind Pit-1 with the same potential as equivalent *hGH-N* promoter sequences to direct expression in the pituitary, and some expression has been detected in transgenic mice. Thus, the lack of hPL gene expression in the human pituitary suggests that their promoters are actively suppressed. P sequences are located upstream of the placental hPL/GH-V genes but not *hGH-N* and are able to repress hGH/PL promoter activity in pituitary cells in vitro. P sequences also interact with the LCR at HS III in human pituitary tissue and are able to support the binding of a complex that includes FOXA1, NF-1 and Pit-1 with pituitary cell repressor activity. FOXA1 can bind DNA independent of chromatin condensation and will facilitate the local recruitment of NF-1. Further interaction with Pit-1 bound to promoter sequences provides pituitary specificity, and the configuration of the promoter and P sequence complex presumably hinders transcription. The question of whether other external factors or posttranslational modification of repressor complex factors play a role or whether co-repressors are required/recruited remains to be determined. Importantly, however, the P sequences remain largely untested in their native context in situ or in a developmentally dynamic system. Thus, while evidence supporting the ability of P sequences to actively repress the hPL/GH-V genes in the human pituitary has grown with new evidence of interaction with the LCR (HS III), it still awaits functional testing.

## Data Availability

The original contributions presented in the study are included in the article, further inquiries can be directed to the corresponding author/s.
