# Correction: Cattini, P.A.; Jin, Y. Evidence for Pituitary Repression of the Human Growth Hormone-Related Placental Lactogen Genes and a Role for P Sequences. *Int. J. Mol. Sci.* 2025, *26*, 4421

**DOI:** 10.3390/ijms262311531

**Published:** 2025-11-28

**Authors:** Peter A. Cattini, Yan Jin

**Affiliations:** Department of Physiology and Pathophysiology, University of Manitoba, Winnipeg, MB R3E 0J9, Canada

The authors wish to make the following corrections to this paper [1]. The Erratum version includes removal of a duplicated statement in Section 1; removal of three paragraphs not related to the manuscript that were included by mistake as a copy/paste error; and importantly, re-alignment of references in the text with the correct references in the bibliography. In terms of scientific content, there are no changes to the figures and none of these corrections affect the figures/results presented in the manuscript or their discussion.

## Error in Abstract

Although there are five hypersensitive sites as originally described (HS I-V), only four play a role in pituitary expression of the human growth hormone gene as HS IV is “placenta-specific”. In addition, “transcription” is corrected to “transcription factors” and “is” is corrected to “are”, reflecting that “factors” is a plural.

## Errors in Section 1

The statement “The hGH/PL genes have proven to be an important and intriguing model for understanding gene regulation, given their structural similarity and expression in two distinct tissues and independent cell lineages.” was duplicated in this section, and the second (duplication) has been removed.

Furthermore, three paragraphs have been removed from the end of Section 1 that are unrelated to P sequences and pituitary repression. These paragraphs described a potential role for a transcription factor called PEG3 in activating the human placental lactogen genes in the syncytiotrophoblast that were included by mistake as a “copy/paste error”.

## Error in References

There is a mis-alignment of the references cited in the text and the bibliography at the end of the manuscript. This likely occurred due to re-positioning of figures, which was not detected during preparation of the manuscript for publication.

The authors state that the scientific conclusions are unaffected. This correction was approved by the Academic Editor. The original publication has also been updated.
